# Acute Adrenal Suppression Following Resistance Training in Elite Female Athletes: A Comprehensive Steroid Profile

**DOI:** 10.3390/sports13120426

**Published:** 2025-12-03

**Authors:** Sabrina Vollrath, Norman Bitterlich, Dennis Lüdin, Adrian Rothenbühler, Anthony C. Hackney, Silvio R. Lorenzetti, Anna Drewek, Basil Achermann, Therina du Toit, Petra Stute

**Affiliations:** 1 Department of Obstetrics and Gynecology, Inselspital, University of Bern, 3010 Bern, Switzerland; 2 Section Performance Sport, Swiss Federal Institute of Sport, BASPO, 2532 Magglingen, Switzerland; dennis.luedin@baspo.admin.ch (D.L.);; 3 “Statistik—Analyse, Beratung, Weiterbildung”, 09114 Chemnitz, Germany; norman.bitterlich@t-online.de; 4 Department of Exercise and Sport Science, University of North Carolina, Chapel Hill, NC 27514, USA; 5 School of Engineering, ZHAW Zurich University of Applied Sciences, 8401 Winterthur, Switzerlandachr@zhaw.ch (B.A.); 6 Department of Health Science and Technology, ETH Zurich, 8006 Zurich, Switzerland; 7 Department of Nephrology and Hypertension, Inselspital, University of Bern, 3010 Bern, Switzerland

**Keywords:** steroid profiling, resistance exercise, adrenal androgens, 11-oxygenated androgens, velocity-based training method

## Abstract

**Aim:** This prospective observational study aimed to evaluate acute adrenal-derived hormonal responses and training performance in elite female athletes during resistance training with respect to the female cycle. **Methods:** In 19 elite female athletes, acute hormonal responses to resistance training were examined over four weeks, measured before and 60 min after exercise. Liquid chromatography–mass spectrometry provided a comprehensive steroid profile, including classical and adrenal-specific 11-oxygenated androgens. Performance metrics were tracked using a velocity-based training method. **Results:** Sixty minutes after resistance training, significant acute changes in steroid hormone concentrations were observed. Levels of 11β-hydroxyandrostenedione (11OHA4) (−0.707 nmol/L; *p* = 0.012, −20.0%), androsterone (−0.201 nmol/L; *p* = 0.049, −14.8%), and dehydroepiandrosterone (DHEA) (−3.813 nmol/L; *p* = 0.006, −17.1%) decreased significantly. The total sum of glucocorticoids, mineralocorticoids, and bioactive androgens decreased. No significant differences in absolute or relative velocity loss and estimated one-repetition maximum were observed, suggesting comparable strength and fatigue across menstrual cycle phases. **Conclusions:** The observed post-exercise decline in glucocorticoids, mineralocorticoids, and androgens such as DHEA indicates a coordinated acute suppression of adrenal steroidogenesis in response to resistance training in female elite athletes. No differences in strength parameters were observed across menstrual cycle phases.

## 1. Introduction

Optimizing performance through a balanced cycle of training and recovery phases is the focus of every athlete. Resistance training has emerged as a crucial component in athletic development, demonstrating significant benefits across various performance metrics in elite athletes [1]. Research has shown that resistance training programs that are properly structured can lead to substantial improvements in muscle strength, power, and sport-specific performance, with effect sizes ranging from small to very large (~0.15–6.80) [2]. Female athletes, in particular, have shown remarkable adaptations to resistance training, with studies documenting significant improvements in strength, power output, and sport-specific skills observed in tennis serve velocity, volleyball performance, and soccer-specific capabilities [3]. These adaptations extend beyond mere strength gains, encompassing important physiological changes, including enhanced fat-free mass, optimized muscle fiber composition, and improved neural activation patterns [4].

In men, metabolic stress (i.e., metabolic load) during strength training is directly related to muscle growth and strength gains. Elevated lactate levels (a marker of metabolic stress), in particular, are seen as a possible trigger for anabolic signaling processes. These processes are associated with increases in testosterone (T), cortisol, and growth hormone (GH) in the blood, which in turn can promote anabolic and metabolic processes and therefore also muscle adaptation [5].

In females, fluctuations in sex hormones during the menstrual cycle may influence the response to exercise training. For example, strength training during the follicular phase may be more effective than training during the luteal phase [6]. However, a recent study examining movement velocity across different exercises, including the bench press, concluded that this parameter remains stable throughout the female menstrual cycle [7]. In both sexes, the scientific interest in the capacity of resistance training to elicit acute hormonal responses, particularly regarding transient changes in anabolic hormones and their metabolites, has grown in recent years [4,8,9,10,11,12,13,14,15,16,17,18,19,20]. Especially in men, several studies have demonstrated a positive effect of strength training on androgenic hormones—most notably T—following resistance training [8,13,21,22,23,24]. However, these findings have not been consistently replicated, with other studies reporting minimal or no changes in hormonal concentrations in male athletes in the acute post-exercise phase [25,26] or after several weeks of resistance training [4,18,27]. In women, the endocrine response to resistance training has been even more variable. Acute responses involving key hormones such as cortisol, T, GH, and insulin-like growth factor 1 (IGF-1) have been examined across a wide range of female populations, differing in training status and age, with the exercise protocol ranging from low-volume single-set circuits to high-volume multiple-set training [11,13,14,15,16,28,29,30,31,32,33,34,35,36,37,38,39,40,41,42,43,44,45,46]. Despite extensive research, it remains unclear whether resistance exercise reliably triggers acute endocrine responses in women, particularly with regard to anabolic androgens. The existing body of literature shows marked inconsistencies, which can be categorized into studies reporting either hormonal changes following resistance training or no hormonal changes. Several investigations have observed acute significant increases in androgens [14,16,47] following resistance training, whereas others show only marginal or statistically non-significant acute elevations in androgen levels [48,49]. Other research has documented sustained elevations in androgen levels, with long-term increases in androgen levels in female athletes [9,15,50], reflecting potential endocrine adaptation over time. By contrast, other studies have found acute or long-term decreases [11]. Nevertheless, a substantial portion of the literature shows no measurable acute or chronic change in androgen levels in response to resistance training in women [4,8,10,12,13,17,18,19,20,28,32,35,51,52,53,54,55]. Moreover, this inconsistent literature raises the question of whether potential hormonal fluctuations influenced by training contribute in a functionally relevant way to cellular adaptations such as protein synthesis and muscle remodeling in the female population.

Most studies examining acute hormonal responses to resistance exercise in women have relied on immunoassays [11,13,14,15,16,28,29,30,31,32,33,34,35,36,37,38,39,40,41,42,43,44,45,46]. However, these methods often show coefficients of variation between 5% and 20%, depending on the hormone and assay conditions. Small fluctuations may therefore fall within analytical variability, which is problematic for hormones present at low concentrations, such as T in women, where assay precision is frequently insufficient due to a lack of specificity [56]. These observations underscore the methodological and physiological complexity involved in assessing hormonal responses to resistance training in women. Liquid chromatography–mass spectrometry (LC-MS) offers a promising alternative to immunoassays with better specificity and sensitivity [57].

Velocity-based training (VBT) has emerged as an effective approach to regulate training intensity for enhancing muscle strength and athletic performance across various populations. Recent research demonstrates that VBT protocols, typically utilizing velocity loss thresholds of 15–30% and intensities of 70–80% of one-repetition maximum (1RM) [58], can lead to significant improvements in strength and performance measures [59]. Studies involving trained female athletes have shown remarkable results, with improvements in one-repetition maximum strength ranging from 8 to 23% [60] and enhanced performance in measures such as countermovement jump and sprint performance [61]. Notably, young females have shown substantial improvements in strength measures, with one study reporting a 28% increase in squat 1RM using VBT compared with traditional training approaches [62]. While most research has focused on trained athletes, emerging evidence suggests that VBT may be effective across different training statuses, though direct comparisons between trained and untrained populations remain limited [63]. In VBT, the velocity of each repetition can be monitored using different devices [64,65]. Velocity has been shown to be related to fatigue, repetitions in reserve, and 1RM, and, as such, allows for monitoring training progress [66].

Therefore, this study aimed to examine the acute endocrine response to resistance training in elite female athletes using comprehensive steroid profiling via LC-MS and, in parallel, to assess strength-related performance parameters using VBT. Furthermore, this research endeavored to address the existing gap in the literature regarding the correlation between VBT approaches and hormonal changes. We hypothesized that resistance training induces acute changes in endocrine markers, particularly androgens and their related metabolites. The primary endpoint of this study was the change in dehydroepiandrosterone (DHEA) post-exercise. Secondary endpoints included changes in other androgens, including novel adrenal markers such as 11KT (11-ketotestosterone), 11OHA4 (11β-hydroxyandrostenedione), 11OHT (11β-hydroxytestosterone), 11KA4 (11-ketoandrostenedione), and performance metrics assessed through VBT-derived parameters, which likewise enable examination of potential menstrual cycle effects.

## 2. Methods

### 2.1. Participants

Nineteen healthy, female elite athletes experienced with resistance training were recruited for this study. Participants were screened based on predefined exclusion criteria, which included chronic diseases, pregnancy, breastfeeding, physical complaints (injuries or ongoing rehabilitation of injuries), and endocrine disorders (e.g., type II diabetes, polycystic ovarian syndrome, and endogenous androgen deficiency). All participants provided both oral and written informed consent after receiving a comprehensive explanation of the study’s objectives, methodology, potential risks, and safety precautions. The Ethics Committee of the canton Bern, Switzerland, reviewed and approved the study protocol (Project-ID: 2024-00895). This study was conducted in full compliance with the ethical principles outlined in the Declaration of Helsinki [67].

### 2.2. Study Design

**Figure 1:**
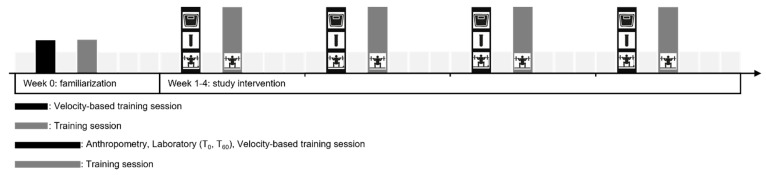
Illustration of the study design.

This was a monocentric, prospective observational study investigating the acute hormonal response to resistance training in elite female athletes. The resistance training was part of the athletes’ regular training schedule and was not modified or prescribed by the study protocol. All participants engaged in resistance training twice a week over a four-week period, with laboratory assessments performed once a week, both before and after training (Figure 1). A one-week familiarization phase preceded the intervention to ensure the standardization of testing procedures and the execution of training. The sample size was estimated based on the sample sizes reported in comparable studies [14,50,68] and an assumption regarding the expected testosterone response, which is subject to uncertainty. Specifically, we assumed that an average increase of 1.25 nmol/L from a baseline of 2.5 nmol/L (Cohen’s *d* ≈ 0.5) would represent a meaningful effect. Based on these calculations, a total of 34 paired pre- and post-training measurements was considered adequate to detect a statistically significant difference at a level of 0.05 with 80% power. Although the power calculation was based on testosterone due to the availability of reference data, DHEA was defined a priori as the primary endpoint according to the approved ethics protocol.

### 2.3. Measurements

#### 2.3.1. Testing Procedures

To evaluate the acute endocrine response after strength training, blood sampling was performed during the first training session of each week over a four-week resistance training protocol, with measurements taken immediately before exercise (T0) and 60 min post-exercise (T60) (Figure 1).

#### 2.3.2. Anthropometric Measurements

Height was measured to the nearest 0.5 cm using a wall-mounted stadiometer (Seca 217, Seca, Hamburg, Germany). Body mass was measured to the nearest 0.1 kg using a medical scale (Seca 803, Seca, Hamburg, Germany).

#### 2.3.3. Estimated One-Repetition Maximum and Intra-Set Velocity Loss of the Back Squat Exercise

Load–velocity profiles were assessed using the average of the mean propulsive velocities (MPVs) and corresponding loads during the back squat exercises [69]. The load for the estimated 1RM for the back squat for each VBT session was determined by fitting a linear regression model and using a velocity threshold of 0.3 m/s for the first VBT session [70,71]. The difference in MPVs between the first and last repetitions within each set was defined as the absolute velocity loss. For each athlete, the velocity losses from all sets performed within a training session were averaged to obtain a single representative value per athlete per session. Relative values were normalized to the maximal mean velocity. A linear encoder unit using a string attached to the barbell was set up for the MPV measurements (Vitruve, Madrid, Spain). An individualized box height was used to standardize the individual depth of the back squat (at least 90° knee angle). The height of the box was not changed during the study period. Participants were encouraged to execute the concentric phase of each repetition with maximal velocity. Estimated 1RM calculations were conducted using Microsoft^®^ Excel^®^ software version 2502 (Microsoft Corporation, Redmond, WA, USA) [72], and calculations concerning velocity loss were conducted using R Statistical Software version 4.5.0 (R Core Team, Vienna, Austria).

#### 2.3.4. Blood Samples and Biochemical Analyses

**Figure 2:**
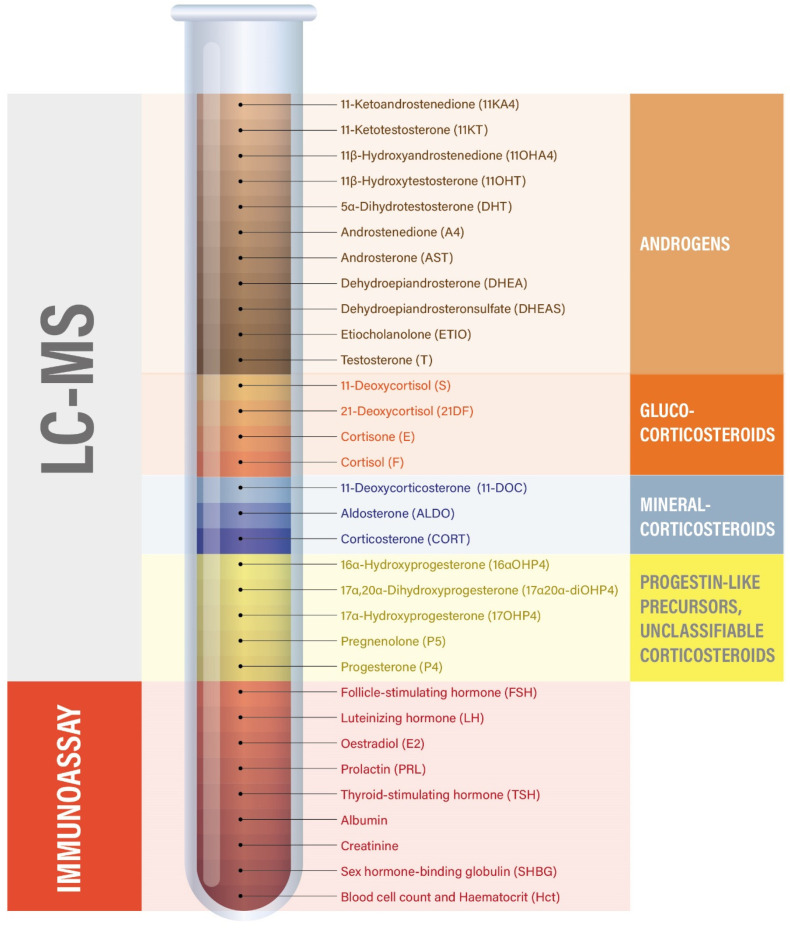
Overview of blood samples and biochemical analyses.

**Figure 3:**
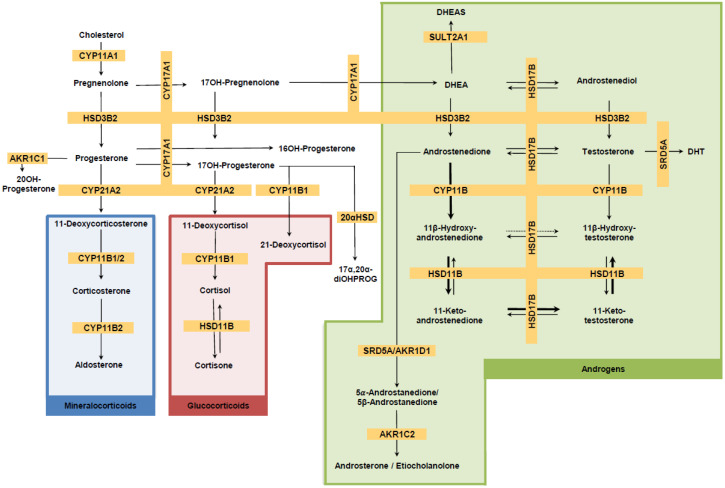
Steroid biosynthetic pathways. **Description:** Steroids in gray were not included in our analysis. Bold arrows show preferred conversion. The dotted arrow represents negligible conversion. See Table 1 for steroid abbreviations. CYP11A1, Cytochrome P450 cholesterol side chain cleavage; 3βHSD2, 3β-Hydroxysteroid dehydrogenase type 2; CYP17A1, Cytochrome P450 17α-hydroxylase,17,20-lyase; CYP21A2, Cytochrome P450 steroid 21-hydroxylase; CYP11B1, Cytochrome P450 11β-hydroxylase; CYP11B2, Cytochrome P450 aldosterone synthase; HSD11B, 11β-Hydroxysteroid dehydrogenase; HSD17B, 17β-Hydroxysteroid dehydrogenase; SRD5A, Steroid-5α-reductase; AKR1D1, aldo-keto reductase family 1 member D1; SULT2A1, sulfotransferase; AKR1C2, aldo-keto reductase family 1 member C2; 20*α*HSD, 20*α*-Hydroxysteroid dehydrogenase.

Venous blood samples were collected on site into EDTA-Plasma tubes before (T0) and 60 min after training (T60) via venipuncture from the antecubital vein. The 60-min post-exercise time point was selected as a compromise between physiological relevance and participant feasibility. Samples were left at room temperature to coagulate for 10 min, after which the samples were centrifuged at 4 °C for 10 min at 1000–2000× *g*. Thereafter, 500 µL of plasma was aliquoted into labeled 1.5 mL microcentrifuge tubes and stored at −20 °C until analysis was initiated. Transport of samples was at room temperature. Steroid profiling (see Figure 2 and Figure 3) was performed using a validated LC-MS method [73]. Briefly, steroids were extracted from plasma after spiking with 38 μL of a mixture of internal standards, a protein precipitation step using zinc sulfate and methanol, and solid-phase extraction with an OasisPrime HLB 96-well plate (Waters Corporation, Milford, MA, USA). Samples were resuspended in 100 μL of 33% methanol in water, and 20 μL was injected into the LC-MS instrument (Vanquish UHPLC coupled to a QExactive Orbitrap Plus, Thermo Fisher Scientific, Waltham, MA, USA) using an Acquity UPLC HSS T3 column (Waters Corporation, Milford, MA, USA). Accuracy and precision have been validated to be below 15% relative standard deviation (RSD) and relative error for both inter- and intra-day validation runs tested at high, medium, low, and lower limit of accurate quantification concentration levels. In addition, analyte recovery was validated as >80% in all cases. Serum estradiol, progesterone, luteinizing hormone (LH), follicular-stimulating hormone (FSH), prolactin, and thyroid-stimulating hormone (TSH) were additionally measured using electrochemiluminescence immunoassays on a Roche Cobas 8000 automated analyzer (Roche, Rotkreuz, Switzerland). Steroid hormone binding globulin (SHBG) was measured with a solid-phase two-site chemiluminescent immunometric assay (Siemens, IMMULITE^®^ 2000 SHBG). Likewise, plasma albumin (bromocresol purple method) and creatinine (enzymatic method) were analyzed on a Roche Cobas 8000 automated analyzer (Roche, Rotkreuz, Switzerland). Additional parameters included complete blood count and hematocrit, which were measured on a Sysmex XR-9000 hematologic analyzer (Sysmex Suisse AG, Horgen, Switzerland). Steroid pathway involvement was estimated by grouping steroids into subcategories based on their biochemical origin (e.g., adrenal-only androgens, ovarian and adrenal androgens, corticosteroids, and mineralocorticoids), summing and weighting their concentrations to approximate the hormonal output of distinct biochemical pathways and tissue sources (see Table 1) [74].

### 2.4. Menstrual Cycle Phase Determination

The individual menstrual cycle phase was determined based on the combination of serum hormones and the calendar-based counting method [75]. Therefore, blood samples were taken before the training session, and participants were additionally asked to report the date of their last menstrual period. Classification into three phases was based on hormone concentrations of luteinizing hormone (LH), progesterone (P4), and estradiol (E2): follicular phase (LH: 2.0–14.0 U/L; P4: <0.700 nmol/L; E2: 80–2000 pmol/L), periovulatory phase (LH: 15.0–96.0 U/L; P4: 1.000–7.000 nmol/L; E2: >500 pmol/L), and luteal phase (LH: 1.0–11.4 U/L; P4: >7.000 nmol/L; E2: 180–800 pmol/L). The classification was based on lab-specific reference ranges. In cases where classification was inconclusive, final assessment was performed by an experienced endocrinological gynecologist.

### 2.5. Resistance Training

The resistance training was performed in a real-world setting. The training program was planned in coordination with coaches and athletes to ensure that it fitted into the athletes’ long-term athletic development as well as short-term competition planning. The first training sessions of each week for each group of athletes are summarized in Table 2. For football athletes, the progression of the MPV and corresponding loads over the four weeks aimed to develop maximum strength in the back squat exercise [60,76]. Other exercises targeting lower body muscle groups focused on power development (i.e., single leg jumps), muscular endurance (i.e., hip thrust), injury prevention (i.e., calf raises and Nordic hamstring curls), and hypertrophy (i.e., exercises targeting the upper body). For track and field athletes, regarding the progressions of repetitions, MPV, and % 1RM, the emphasis of the four-week training program was on maximum strength development [60,76,77]. Other exercises focused on performance over a longer set duration (i.e., hip thrust and exercises targeting the upper body) and sports-specific injury prevention (i.e., eccentric leg press and calf raises).

### 2.6. Statistical Analysis

All parameters were analyzed descriptively. For continuous data, the statistical characteristics are specified: mean, standard deviation (SD), median, quartiles, and extrema. Frequency tables were used for ordinal and nominal variables. Changes over time (pre–post) were evaluated using non-parametric Wilcoxon *t*-tests (indicated by *wil*). The *t*-test was also used for sensitivity analysis. The Mann–Whitney U test (indicated by *U*), also supplemented with a *t*-test, was used to compare the two subgroups. The comparison of data from the four visits was evaluated using the Friedman test. The effect sizes according to Cohen’s d (d) were calculated for changes and for group comparisons. To account for hemoconcentration effects, post-exercise laboratory results were normalized to pre-training values based on the individual change in hematocrit. Missing data within a participant’s visit were replaced with the mean of their available values. Correlations were calculated non-parametrically using Spearman’s rho, indicated by SR. Since only DHEA was defined as the primary endpoint, all other hormonal analyses were considered exploratory; therefore, *p*-values are presented unadjusted and should be interpreted descriptively.

**Weighted profile analysis.** A weighted profile analysis was applied to obtain a physiologically meaningful indicator of pathway-specific steroidogenic activity. Hormonal changes within each pathway were normalized to the concentration scale of the most abundant steroid using linear regression models, whose coefficients served as weighting factors for aggregating pathway-specific responses (see Supplementary Materials File S1). This regression-based weighting was preferred over conventional multivariate methods due to small sample size and non-normal data distribution, ensuring both physiological interpretability and statistical robustness. All hormones within each pathway were aggregated to quantify the pathway-specific response [74,78,79].

To compare velocity loss between the follicular and luteal phases in both football and track and field, Wilcoxon rank sum exact tests were conducted. Comparisons were made for both relative and absolute velocity loss between the two phases. Statistical significance was set at *p* < 0.05, but without consideration of multiple testing, and analyses were performed using SPSS version 19.0 software (SPSS Inc., Chicago, IL, USA) [80].

## 3. Results

### 3.1. Participant Characteristics

Nineteen healthy elite female athletes (n = 19) participated in the study, including eight elite football players (mean age: 18.9 ± 0.8 years) and eleven track and field athletes (mean age: 25.0 ± 3.5 years). A summary of subject demographic characteristics is presented in Table 3. After the completion of the 4-week training program, no significant changes were found in body weight for all elite female athletes (p_wil_ = 0.078). Seven out of nineteen athletes reported the use of hormonal contraception, including combined oral contraceptive pills (n = 2), hormonal intrauterine devices (IUDs) (n = 4), and a copper IUD (n = 1). No cases of amenorrhea were present at the time of data collection. Menarche occurred between ages 10 and 15 in 16 participants (84.2%), while 3 reported onset after age 15 (15.8%), meeting the criterion for primary amenorrhea and consistent with previous findings in elite female athletes [81]. The two groups differed significantly in weekly training volume, with footballers training fewer hours per week than track and field athletes (*p* < 0.001).

### 3.2. Hormonal Responses to Resistance Training

In our study, we profiled 19 elite female athletes and compared their steroid panels pre- and post-training. In total, twenty-three steroids were quantified using the LC-MS method, and changes were compared from pre-training to 60 min post-training. In addition to absolute differences in steroid concentrations, we also calculated the relative changes (post-/pre-ratio) to account for inter-individual baseline variation and to better characterize proportional hormonal shifts induced by acute resistance exercise, as summarized in Table 4 and Table S1.

#### 3.2.1. Acute Post-Exercise Alteration in the Composite Androgen Profile

While not all androgens demonstrated significant changes, several trends were observed with a significant decrease in 11OHA4 post-exercise (−0.707 nmol/L; *p* = 0.01; effect size *d* = 0.65; −21.1%; *p* = 0.01; *d* = 0.64), a significant increase in 11KA4 (+0.079 nmol/L; *p* = 0.023; *d* = 0.57; +24.3%), a significant decrease in AST (−0.201 nmol/L, *p* < 0.05; *d* = 0.34, −14.8%), a significant decrease in DHEA (−3.813 nmol/L; *p* = 0.006; *d* = 0.60, −17.1%), a non-significant increase in DHEAS (+350.700 nmol/L, *p* = 0.145, *d* = 0.22, +9.9%), a non-significant small absolute and relative decrease in ETIO (−0.129 nmol/L; *p* = 0.490, *d* = 0.29, −4.9%), and a statistically non-significant reduction in total T (−0.046 nmol/L; *p* = 0.080; *d* = 0.41, −6.2%). To guess the total “androgen activity/response”, a sum of all androgenic hormones was built (Table 4) out of 11OHA4, 11OHT, 11KA4, 11KT, dihydrotestosterone (DHT), androstenedione (A4), androsterone (AST), DHEA, dehydroepiandrosterone sulfate (DHEAS), T, and etiocholanolone (ETIO). Summing the concentrations of these 11 androgenic hormones did not yield a statistically significant absolute change (+345.000 nmol/L; SD ±1582.500 nmol/L, *p* = 0.145, *d* = 0.22) (Table 3), primarily due to high variability driven by high circulatory concentration hormones such as DHEAS, which is strongly correlated with the total sum (r = 0.998; pSR < 0.001), and due to opposing directional changes among individual hormones. Using the sum of the relative change, a trend toward significance could be shown (+9.7%, SD ± 18.9%, *p* = 0.055, *d* = 0.51). The weighted androgen hormone profile (11OHA4, 11OHT, 11KA4, 11KT, DHT, A4, AST, DHEA, DHEAS, T, and ETIO) showed a significant increase shift post-training (*p* = 0.002, *d* = 0.86)—based on a linear regression model—indicating a hormonal reaction in female elite athletes. When excluding DHEAS due to its negligible bioactivity [82], a significant decrease was observed in the total androgen profile (−4.996 nmol/L, *p* = 0.012, *d* = 0.61), driven by the acute reduction in DHEA.

#### 3.2.2. Acute Post-Exercise Alteration in Adrenal-Derived Androgens

Considering the entire androgen profile, it is worth examining the androgens that originate exclusively from the adrenal glands, mainly 11OHA4, 11OHT, 11KA4, 11KT, and DHEAS (Table 1). The unweighted sum of these hormone concentrations increased non-significantly post-exercise (+349 nmol/L; *p* = 0.145; *d* = 0.22, +9.8%). The dominating steroid DHEAS showed a comparable non-significant increase (+349.800 nmol/L, *p* = 0.145). Significant changes were observed for 11OHA4 (−0.707 nmol/L; *p* = 0.012) and 11KA4 (+0.079 nmol/L; *p* = 0.023) (Table 4). These divergent trends within individual androgens reflect the interconnected nature of steroid biosynthesis. When applying a weighted summation model, statistical significance was reached and the effect size increased, supporting the tendency of an increase in androgens post-training, which exclusively originates from the adrenal glands (*p* < 0.001; *d* = 1.21) (Table 4). For DHEA, which originates from both the adrenal glands and the ovaries (theca cells), a significant decrease (−3.813 nmol/L; *p* = 0.006) was observed (Table 4).

#### 3.2.3. Acute Post-Exercise Alteration of Androgen Metabolites Depending on the Androgen Biosynthesis Pathway

Androgen metabolites were further categorized and examined according to their respective androgen biosynthetic pathways (Table 1), including the classic androgen pathway, the 11-oxy pathway, both the 11-oxy pathway and its direct precursors, and the backdoor pathway. The arithmetic sum of the classic androgen pathway, consisting of six metabolites (DHEA, A4, AST, ETIO, T, and DHT), showed a significant absolute decrease in total androgen concentrations post-exercise (−4.515 nmol/L; *p* = 0.005, *d* = 0.66) and a significant relative decrease (−15.4%; *p* = 0.005; *d* = 0.78). The primary androgen precursors DHEA (−3.813 nmol/L; *d* = 0.006) and A4 (−0.260 nmol/L; *p* = 0.080) contributed substantially to this reduction (Table 4). A weighted summation through linear regression amplified the effect size significantly (*p* < 0.001, *d* = 1.72), confirming the acute downregulation of the classic androgen biosynthesis pathway in response to resistance training. In the 11-oxy pathway, the simple sum of six metabolites (11OHA4, 11OHT, 11KA4, 11KT, A4, and T) did not reach significance due to opposing directional changes (−0.787 nmol/L; *p* = 0.096). However, the weighted profile revealed a significant post-exercise decline (*p* = 0.001; *d* = 0.97) (Table 4), primarily driven by the marked decrease in 11OHA4 (*p* = 0.012). In the backdoor pathway (17OHP4, DHT, AST, and P4), the unweighted profile showed no significant absolute change (+0.040 nmol/L; *p* = 0.953), again reflecting divergent hormone dynamics. Nevertheless, the weighted summation resulted in a statistically significant decline (*p* = 0.007; *d* = 0.72) (Table 4).

#### 3.2.4. Acute Post-Exercise Alteration in Adrenal 11-Oxygenated Adrenal Steroids

11-oxygenated steroids are presented in order of their recognized biosynthesis through the 11-oxy-pathway (first: 11OHA4; second: 11KA4, third: 11KT; and fourth: 11OHT). Among these steroids, the first metabolite, 11OHA4, decreased significantly 60 min post-training in the total group (−0.707 nmol/L, SD = 1.08; *p* = 0.012; *d* = 0.65), whereas the second metabolite, 11KA4, increased significantly (+0.079 nmol/L; SD = 0.13; *p* = 0.023; *d* = 0.57) and the other metabolites, 11OHT and 11KT, showed no significant alterations (Table 4). The unweighted sum of these four metabolites showed no significant changes (*p* = 0.134). Due to the individual strongly intercorrelated hormonal levels, a weighted approach revealed a significant overall decline (*p* = 0.002, *d* = 0.92), reflecting a coordinated pathway-specific and, here especially, adrenal-specific acute response to acute resistance exercise.

#### 3.2.5. Acute Post-Exercise Alteration in Glucocorticoids and Mineralocorticoids

The glucocorticoid (11-deoxycortisol, 21-deoxycortisol, cortisol, and cortisone) and mineralocorticoid (11-deoxycorticosterone, corticosterone, and aldosterone) profiles (Table 1) exhibited consistent and statistically significant post-exercise declines in each hormone (Table 4). This was reflected in a significant overall reduction in total glucocorticoid concentrations (−107.390 nmol/L; *p* < 0.001; *d* = 1.22) and total mineralocorticoid concentrations (−5.058 nmol/L, *p* = 0.001, *d* = 0.87) (Table 4). The most pronounced absolute decrease was observed in cortisol (−102.720 nmol/L, −30.9%, *p* < 0.001; *d* = 1.11) and contributed strongly to the overall decline in glucocorticoids. Within the mineralocorticoids, corticosterone emerged as the leading hormone in both absolute and relative terms (−5.010 nmol/L, *p* = 0.001, *d* = 0.87). The weighted summation model further increased the effect sizes for both glucocorticoids (*p* < 0.001, *d* = 2.12) and mineralocorticoids (*p* < 0.001, *d* = 1.47), indicating a coordinated suppression of adrenal glucocorticoid and mineralocorticoid steroid output in response to resistance training.

#### 3.2.6. Hormonal Concentrations and Hormonal Responses Across the Menstrual Cycle

For group comparisons, participants of the first week of measurements were categorized according to their menstrual cycle phase into a “follicular phase group” (P4 < 1.000 nmol/L; n = 15) and a “luteal phase group” (P4 > 5.000 nmol/L; n = 2). Participants using oral contraceptives (n = 2) were excluded from this analysis. In the “luteal phase group”, higher concentrations were observed for metabolites downstream of progesterone, specifically 11-DOC (*p* = 0.019), 16αOHP4 (*p* = 0.038), 17α20α-diOHP4 (*p* = 0.019), 17OHP4 (*p* = 0.019), 20α-hydroxyprogesterone (*p* = 0.019), P5 (*p* = 0.067), and P4 (*p* = 0.019). Regarding hormonal changes from pre- to post-resistance training, P5 was the only hormone that showed a significant decrease in the first measurement week (visit 1) (−0.299 nmol/L, *p* = 0.038) (see Supplementary Table S2). However, this pattern was not replicated in subsequent weeks (visits 2–4). Consequently, the menstrual cycle phase was not further considered in the statistical analysis of hormonal changes.

#### 3.2.7. Alterations in Additional Hormonal-Related Parameters

SHBG levels showed a non-significant reduction from pre- to post-training over the four-week period (*p* = 0.065), with no significant differences across pre- or post-training time points or between athlete groups. Prolactin levels were not statistically different between the four weeks (*p* = 0.823). LH, FSH, and estradiol levels were determined before the training (T0) over the four-week training period, and supported the categorization of the athletes into their menstrual cycle phases. However, progesterone levels, measured via immunoassay, served as the main hormonal parameter for categorizing the athletes into either the follicular (<0.700 nmol/L) or luteal (>0.700 nmol/L) phases at T0. TSH levels (T_0_) were measured within the normal reference range for all athletes over the four-week training period. A statistically significant change in albumin levels was observed from pre- to post-training (Z = −2.360, *p* = 0.016), reflecting a slight overall increase in mean values (from 40.5 g/L to 41.3 g/L). Creatinine levels did not differ among the four pre-measurements (T_0_) over the four-week training period (*p* = 0.839).

### 3.3. Velocity-Based Training Measures

No significant changes in movement velocity or estimated 1RM were observed over the 4-week intervention. Menstrual cycle phase-related differences in relative (football: W = 18, *p* = 0.25; track and field: W = 17, *p* = 0.14) and absolute (football: W = 19, *p* = 0.17; track and field: W = 20, *p* = 0.25) velocity loss were not significant. No phase-related difference was observed for the estimated 1RM or the load–velocity profile.

## 4. Discussion

In the current study, we show that resistance training in healthy elite female athletes leads to a significant and coordinated acute decrease in adrenal-synthesized steroid hormones, including glucocorticoids, mineralocorticoids, and classic and 11-oxygenated androgens such as DHEA (DHEA) (−3.813 nmol/L; *p* = 0.006, −17.1%) and 11OHA4 (−0.707 nmol/L; *p* = 0.012, −20%). Hormonal responses were interpreted and characterized by classifying steroids into biochemical subgroups: androgenic steroids, exclusively adrenal-synthesized androgens, mineralocorticoids, and glucocorticoids. In addition, classification was based on the known biosynthetic pathways (the classic pathway, the 11-oxy pathway, both the 11-oxy pathway and its direct precursors, and the backdoor pathway) to enable a more comprehensive analysis of the adrenal steroidogenic response following resistance training. The findings (the decrease in DHEA, the predefined primary endpoint) are statistically robust, whereas analyses of other hormones and pathway profiles were exploratory and not confirmatory.

### 4.1. Post-Exercise Change in Androgens

Resistance training induced a complex, pathway-specific modulation of circulating androgens. Individual androgens showed divergent responses, with significant decreases in DHEA, 11OHA4, and androsterone but a significant increase in 11KA4. The overall unweighted androgen sum remained unchanged, likely due to opposing trends and high variability driven by DHEAS. In contrast, the weighted profile analysis revealed significant pathway-specific declines, particularly within the classic and 11-oxyandrogen pathway, indicating a coordinated downregulation of adrenal steroidogenesis following resistance training. These findings highlight that the acute androgen response in elite female athletes is a multidimensional and integrated response rather than an individual increase or decrease in circulating androgens.

The literature on the acute androgen response to resistance training in women remains inconsistent. While several studies have reported significant post-exercise increases in androgen concentrations [14,16,47], others found only marginal or statistically non-significant acute elevations [48,49], and some even observed transient decreases [11]. However, the majority of studies reported no measurable change in circulating androgens following resistance exercise [4,8,10,12,13,17,18,19,20,28,32,35,51,52,53,54,55].

These discrepancies may, at least in part, be attributed to differences in sampling time, training modality, and exercise duration. A recent meta-analysis on high-intensity interval training (HIIT), which represents a distinct exercise modality from resistance training, showed that both testosterone and cortisol levels increase immediately after exercise but return to or drop below baseline within 60 min [83]. Thus, the blood sampling at 60 min post-exercise in our study likely reflects the late phase of the acute endocrine response, capturing recovery from an earlier transient peak of adrenal steroidogenic activity. Earlier studies in male athletes have shown that stress-related hormones such as cortisol and testosterone typically peak within the first 30 min after exercise and then progressively return toward baseline within 180 min [84]. Methodological differences may also contribute to inconsistencies across studies. Earlier investigations in women predominantly relied on immunoassays [11,13,14,15,16,28,29,30,31,32,33,34,35,36,37,38,39,40,41,42,43,44,45,46], which have limited sensitivity to reliably quantify changes in androgens at the low physiological concentrations typical for females.

Overall, the acute androgen response was strongly characterized by the dominance and acute decline in DHEA concentrations. DHEA is predominantly synthesized in the zona reticularis of the adrenal cortex and, to a lesser extent, in the ovary. As an early product of the classic androgen pathway, it is derived from cholesterol via pregnenolone and subsequently converted by cytochrome P450 17A1 (CYP17A1) into DHEA [85,86]. DHEA is therefore a steroid hormone in the sense that it is produced rapidly in response to physiological stress, such as cortisol and corticosterone. Although DHEA primarily acts as a prohormone for more potent androgens such as testosterone and DHT, its rapid regulation makes it a sensitive marker of adrenal steroidogenic activity and acute physiological stress. Interestingly, few studies have been conducted on the dynamics of DHEA after resistance training. Some previous studies have reported a decrease in DHEA one hour after resistance training, but it was not statistically significant [87], while others found an acute increase in DHEA [14,40,47,68,88,89]. In addition to the acute decrease in DHEA as a result of resistance training in our study, another aspect of DHEA is worth mentioning. The growing body of evidence suggests that DHEA plays a role in modulating oxidative stress. It is well-documented that regular resistance training reduces the risk of developing various types of cancer (e.g., breast cancer) and thereby exerts a preventive effect on tumorigenesis. Although DHEA acutely decreases following resistance training, regular resistance training is generally assumed to lead to a long-term increase in basal levels of IGF-1, SHBG, and DHEA in both males and females over 40 years of age [90]. DHEA, in turn, has antioxidant properties. It acts as a non-competitive inhibitor of glucose-6-phosphate dehydrogenase (G6PD), thereby reducing the availability of NADPH. Since NADPH is a central substrate for the activity of NADPH oxidases (NOX), this inhibition leads to reduced production of reactive oxygen species (ROS) [91]. In this way, repeated secretion of DHEA could contribute to cancer-preventive [92], anti-inflammatory, and immunomodulating effects that reduce the risk of age-related diseases such as cancer, atherosclerosis, and neurodegenerative disorders.

### 4.2. Post-Exercise Change in Glucocorticoids

In this study, a coordinated and statistically significant decrease in both glucocorticoids and mineralocorticoids was observed following resistance training.

Cortisol showed the most pronounced absolute decline (−102.700 nmol/L, −30.9%, *p* < 0.001), contributing substantially to the overall reduction in total glucocorticoids (−107.300 nmol/L, *d* = 1.22). Within the mineralocorticoids, corticosterone demonstrated the strongest decrease (−5.01 nmol/L; *p* = 0.001), driving the observed reduction in total mineralocorticoids (*d* = 0.87). The large effect sizes in the weighted summation model (*d* = 2.12 for glucocorticoids; *d* = 1.47 for mineralocorticoids) point to a systemic suppression of adrenal steroid output in response to acute resistance exercise. These findings are in line with previous research on hormonal responses to resistance training in women. In a controlled crossover study, resistance-trained women performed hypertrophy (70% 1RM), strength (90% 1RM), and power-type (45% 1RM) protocols. All modalities led to a significant post-exercise decrease in serum cortisol (*p* < 0.05), with the hypertrophy protocol eliciting the most pronounced endocrine response [93], which has also been confirmed in other studies [94]. Similar cortisol decreases 60 min post-exercise have been reported by Nakamura et al. [43] in female athletes across different menstrual phases, as well as in middle-aged inactive women, where an approximate 17% decline in cortisol was observed immediately after resistance training [95] and likewise in resistance-trained women [93]. Although testosterone levels in women often remain unchanged following resistance training, the concurrent acute decrease in cortisol leads to an increase in the testosterone-to-cortisol ratio, which has been described in other studies as indicative of a more anabolic environment [93]. Other studies showed a cortisol spike 15 [48,94], 30 [48], or 60 min after resistance exercise [96]. Uchida et al. reported an acute post-exercise decrease in cortisol, but more importantly, a long-term reduction in resting cortisol levels as an adaptation to repeated training stimuli, resulting in an increased testosterone-to-cortisol ratio [97].

### 4.3. Post-Exercise Change in Mineralocorticoids

The zona glomerulosa of the human adrenal cortex is the site of biosynthesis of the main mineralocorticoid aldosterone [98]. Aldosterone plays a crucial role in maintaining fluid and electrolyte balance, as well as regulating blood pressure through sodium (Na^+^) retention. Its secretion by the adrenal cortex is regulated via the renin–angiotensin–aldosterone system (RAAS). Physical stressors such as resistance training activate the RAAS through increased sympathetic activity and renal hypoperfusion, leading to renin release, the formation of angiotensin II, and subsequent aldosterone secretion [99]. In addition, aldosterone secretion is triggered by neuroendocrine stress signals during resistance training through the hypothalamic–pituitary–adrenal (HPA) axis, where corticotropin-releasing hormone (CRH) from the hypothalamus triggers the release of adrenocorticotropic hormone (ACTH) in the pituitary, which enhances aldosterone production in the adrenal. The time course of mineralocorticoid changes, particularly aldosterone, has been well-documented [100]. Studies in male athletes show a rapid activation of the RAAS during resistance training, with elevated aldosterone concentrations occurring within 10–15 min and returning to baseline within 30–60 min post-exercise [100]. However, data on the acute mineralocorticoid response to resistance training in female athletes are limited. Our analysis 60 min post-exercise revealed a significant decline in both glucocorticoid and mineralocorticoid steroid concentrations. Specifically, the mineralocorticoid profile, including 11-deoxycorticosterone, corticosterone, and aldosterone, showed a consistent and statistically significant decrease, with corticosterone demonstrating the most pronounced absolute and relative reduction (−5.010 nmol/L; *p* = 0.001; *d* = 0.87) 60 min post-training. These results align with the reported time frame in which aldosterone typically returns to baseline after acute physical stress [100]. While most previous studies have focused on aldosterone alone and were conducted in male athletes [100], our data implicate a broader decrease in the mineralocorticoid steroid pathway 60 min post-training. Our findings, showing a decrease in mineralocorticoid concentrations 60 min post-training, may reflect the recovery phase of adrenal steroid regulation in female athletes. This aligns with previous studies indicating that mineralocorticoids, particularly aldosterone, rise acutely during resistance training due to RAAS activation but return to baseline following exercise cessation [101].

### 4.4. Coordinated Adrenal Steroid Suppression After Resistance Training

The observed concurrent suppression of adrenal steroids (glucocorticoids, mineralocorticoids, and selected adrenal androgens) suggests a coordinated downregulation of adrenal steroidogenesis rather than isolated changes in individual hormones. The post-exercise values in our data likely represent the early recovery phase after initial activation of adrenal steroidogenesis. The initial brief activation of the HPA axis and the sympathetic adrenomedullary system facilitates energy substrate mobilization, immune signaling, and tissue remodeling [102], before adrenal steroid hormones rapidly drop down and return slowly toward baseline concentrations. Our finding of decreases in multiple adrenal steroids in our study is therefore consistent with this recovery mechanism of adrenal steroidogenesis after exercise training [103].

### 4.5. Hormonal Concentrations and Hormonal Response Across the Menstrual Cycle

Although absolute concentrations of progesterone and its physiological downstream metabolites (11-DOC, 16αOHP4, 17α20α-diOHP4, 17OHP4, 20α-hydroxyprogesterone, and P5) were significantly higher during the luteal phase, this was not associated with overall differences in hormonal reactivity. The change in pregnenolone concentrations following resistance training differed significantly between the luteal (n = 2) and follicular phase groups (n = 15) during the first measurement week, but this effect was not confirmed in subsequent weeks. Therefore, no menstrual cycle phase-dependent modulation of the acute hormonal response to resistance training was observed. Although the study design allowed for the exploratory assessment of menstrual cycle effects, the sample size was not primarily powered for these subgroup comparisons and should be interpreted with caution.

### 4.6. Mechanism of Hormonal Change

The above-mentioned coordinated decline in adrenal steroids in this study likely reflects an integrated recovery response of the HPA axis following acute activation. Beyond the regulatory feedback within the HPA axis, which likely mediates the coordinated suppression observed, other molecular pathways might also contribute. However, we speculate that one possibility is via the upregulation of the Wnt/β-catenin signaling pathway [104], as this pathway has been shown to inhibit adrenal steroidogenesis in vitro [105]. Concurrently, resistance exercise training is known to increase muscle Wnt/β-catenin activity (i.e., content) [106]. Granted, though, research on the Wnt/β-catenin signaling pathway and exercise has almost exclusively been studied in skeletal muscle tissue. Our participants were elite female athletes who were experienced in resistance training regimens; hence, whether such a hormonal response would be observed in untrained individuals is unclear. However, it is well-recognized that chronic exercise training results in reduced adrenal glucocorticoid responses [107] to an exercise session with a given workload as an adaptation in the endocrine stress system, allowing for more efficient recovery [108]. In addition to adrenal steroids, other anabolic hormones such as growth hormone (GH) and insulin-like growth factor 1 (IGF-1) are modulated by resistance training and may further contribute to the adaptive response, particularly in women [33,109]. While androgen levels typically rise acutely but become blunted after training for hours, GH and IGF-1 have been shown to increase transiently after high-intensity resistance training and may play a key compensatory role in muscle adaptation and remodeling [33,109]. Although transient hormonal elevations and mechanical load may theoretically induce short-term upregulation of cytoplasmic or nuclear hormone receptors, such as the androgen receptor (AR) [110], current evidence suggests that this mechanism is not strongly linked to hypertrophy outcomes [111]. Instead, intracellular signaling pathways such as mTOR activation, satellite cell recruitment, and muscle-specific gene transcription appear to play a more decisive role in mediating skeletal muscle adaptation [112]. It seems that muscle adaptation in women relies on multiple coordinated anabolic pathways beyond steroid androgens alone.

Finally, adrenal steroid hormones can be viewed as part of the components of the internal milieu that create a positive environment for facilitating androgenic influences of skeletal muscle plasticity, which is critical for the athlete to improve performance. That said, the decline in adrenal steroid hormones post-exercise could be viewed as counterproductive. However, exercise training, particularly resistance training, can upregulate the internal mTOR signaling pathway and enhance skeletal muscle anabolic activity, thereby promoting alternative means of adaptive responses within the muscle, as reported in the literature [113].

### 4.7. Intra-Set Velocity Measures of the Back Squat Exercise and Hormonal Changes During Menstrual Cycle

While the luteal phase is characterized by elevated progesterone and comparatively lower estrogen concentrations, our findings showed no menstrual cycle phase-specific differences in estimated 1RM or velocity loss, suggesting that menstrual cycle phase did not affect performance in this cohort. Based on the elevated progesterone and reduced estrogen levels during the luteal phase, which may impair neuromuscular efficiency and increase perceived exertion [114,115], a phase-specific difference would have been expected. However, velocity–force profiling during strength testing seems not to be affected by the female cycle [116]. A recent systematic review by Colenso-Semple et al. observed mixed findings across several studies, with no consistent pattern on whether menstrual cycle phase affects performance and hormonal response, whereby menstrual cycle verification was methodologically insufficient in the majority of cases [117]. Hence, our results underscore the importance of further investigating how to integrate menstrual cycle tracking into the athletic programming of female athletes. Whether such integration improves performance and recovery remains unclear, as current evidence is inconsistent and requires further assessment.

### 4.8. Methodological Considerations

This study applied the gold-standard LC-MS method to accurately quantify low-concentration steroid hormones, which is particularly important in female subjects [118]. To our knowledge, only a few studies have investigated the acute response to DHEA following resistance training. Moreover, this is the first study to perform comprehensive steroid profiling in female athletes, allowing for a broader and more integrated interpretation of the acute adrenal response one hour after resistance training. We further included two participants using combined oral contraceptives (COCs), as current evidence indicates that COCs typically result in blunted rather than amplified endocrine responses. Therefore, a general exclusion was not deemed necessary according to current data [119]. A separate subgroup analysis excluding COC users (n = 17) confirmed the robustness of our results, demonstrating that the overall findings remained unchanged (see Supplementary Table S1).

### 4.9. Limitations

Although no bioimpedance analysis (BIA), dual-energy X-ray absorptiometry (DEXA), or CT-based assessment of body composition was performed, the potential effects of plasma volume shifts were controlled for through hematocrit correction for all hormone concentrations. Since the football players were younger, less experienced in resistance training, and trained less than the track and field athletes, a potential association between age, training load, and hormonal responses could not be analyzed in this study and should be addressed in future studies, to also clarify the potential benefits of early resistance training in young female athletes. Furthermore, the comparison across menstrual cycle phases was underpowered due to the small number of participants in the luteal phase and should therefore be interpreted as exploratory. Future studies with larger sample sizes and extended time-course analyses after resistance training are warranted to better characterize steroid dynamics and performance-related effects such as VBT metrics across different menstrual cycle phases.

## 5. Conclusions

Applying comprehensive steroid profiling 60 min post-training using LC-MS in a cohort of elite female athletes, we demonstrated a coordinated decline in adrenal-derived steroids, including androgens (notably DHEA), glucocorticoids (cortisol), and mineralocorticoids (corticosterone), suggesting a post-exercise suppression or recovery phase of adrenal steroidogenesis. However, analysis of VBT data showed no differences in velocity metrics, indicating no menstrual phase-specific effects on strength or fatigue.

## Figures and Tables

**Table 1:** Classification of hormones in biochemical subgroups.

Metabolite	Androgens (Exclusively Adrenal Androgens Marked by xx)	Miner- Alocorti-coids	Gluco- Corticoids	Progestins, Unclassifiable Steroids	Classical Pathway	11-oxy Pathway	11-oxy- Pathway and Its Direct Precursors	Backdoor Pathway
**11-DOC**		x						
**S**			x					
**11KA4**	xx					x	x	
**11KT**	xx					x	x	
**11OHA4**	xx					x	x	
**11OHT**	xx					x	x	
**16αOHP4**				x				
**17α20α-diOHP4**				x				
**17OHP4**				x				x
**21DF**			x					
**DHT**	x				x			x
**ALDO**		x						
**A4**	x				x		x	
**AST**	x				x			x
**E**			x					
**CORT**		x						
**F**			x					
**DHEA**	x				x			
**DHEAS**	xx							
**ETIO**	x				x			
**P5**				x				
**P4**				x				x
**T**	x				x		x	

Abbreviations: 11-DOC = 11-Deoxycorticosterone; S = 11-Deoxycortisol; 11KA4 = 11-Ketoandrostenedione; 11KT = 11-Ketotestosterone; 11OHA4 = 11β-Hydroxyandrostenedione; 11OHT = 11β-Hydroxytestosterone; 16αOHP4 = 16α-Hydroxyprogesterone; 17α20α-diOHP4 = 17α,20α-Dihydroxyprogesterone; 17OHP4 = 17α-Hydroxyprogesterone; 21DF = 21-Deoxycortisol; DHT = 5α-Dihydrotestosterone; ALDO = Aldosterone; A4 = Androstenedione; AST = Androsterone; E = Cortisone; CORT = Corticosterone; F = Cortisol; DHEA = Dehydroepiandrosterone; DHEAS = Dehydroepiandrosteronsulfate; ETIO = Etiocholanolone; P5 = Pregnenolone; P4 = Progesterone; T = Testosterone. Note: “x” indicates hormonal subgroup assignment; “xx” indicates exclusively adrenal androgens.

**Table 2:** Description of the first training sessions of each week, split by sport.

Week	1				2				3				4			
*Football*	Sets	Reps	MPV (m/s)	% 1RM	Sets	Reps	MPV (m/s)	% 1RM	Sets	Reps	MPV (m/s)	% 1RM	Sets	Reps	MPV (m/s)	% 1RM
Back squat	4	4, 4, 3, 3	0.6–0.9		4	4, 3, 3, 2	0.4–0.7		4	3, 3, 2, 2	0.4–0.7		4	3, 2, 2, 2	0.4–0.6	
Single-leg jumps	3	4			3	5			3	6			3	6		
Hip thrust	3	17		60	3	18		60	3	19		60	3	20		60
Eccentric single-leg calf raises	3	10			3	10			3	10			3	10		
Nordic hamstring curls	4	4			4	5			4	6			4	6		
Push-up	4(CS)	6, 8, 10, 12			4	6, 8, 10, 12			4	6, 8, 10, 12			4	6, 8, 10, 12		
Pull-up (with elastic band)	4(CS)	4			4	4			4	4			4	4		
Dips	4(CS)	8			4	8			4	8			4	8		
Incline pull-up	4(CS)	4			4	4			4	4			4	4		
*Track and field*																
Overhead squat	4	6		60	4	6		60	4	6		60	4	6		60
Hang power clean	4	4, 4, 2, 2		80–85	4	4, 3, 2, 2		80–85	5	3, 3, 2, 1, 1		85–90	5	3, 2, 1, 1, 1		90–95
Back squat	4	4, 4, 3, 3	0.4–0.7		4	4, 3, 3, 2	0.4–0.7		4	3, 3, 2, 2	0.4–0.7		4	3, 2, 2, 2	0.4–0.6	
Single-leg step up	4	6, 6, 4, 4		80–85	4	6, 4, 4, 3		80–85	4	4, 4, 3, 3		85–90	4	4, 3, 3, 2		85–90
Eccentric single-leg press	3	5, 4, 3		100–105	3	4, 4, 3		105–110	3	4, 3, 2		105–110	3	3, 3, 2		110
Hip thrust	4	14		60	4	16		60	4	18		60	4	20		60
Eccentric leg press calf raises	3	8		100	3	8		100	3	8		100	3	8		100
Bench press	5(CS)	3, 4, 5, 6, 7		70	5(CS)	3, 4, 5, 6, 7		70	5(CS)	3, 4, 5, 6, 7		70	5(CS)	3, 4, 5, 6, 7		70
Push-up with dumbbells	5	12			5	12			5	12			5	12		
Pull-up with elastic bands	5(CS)	3, 4, 5, 6, 7			5(CS)	3, 4, 5, 6, 7			5(CS)	3, 4, 5, 6, 7			5(CS)	3, 4, 5, 6, 7		

**Note.** Exercises without MPV or % 1RM were performed as bodyweight exercises. CS: cluster set (15 s rest); Reps: repetitions; MPV: mean propulsive velocity; 1RM: one-repetition maximum.

**Table 3:** Subject characteristics.

Characteristic	Football (n = 8) (Mean, ± SD, Median (1st Quartile, 3rd Quartile))	Track and Field (n = 11) (Mean, ± SD, Median (1st Quartile, 3rd Quartile))	Total Group (n = 19) (Mean, ± SD, Median (1st Quartile, 3rd Quartile))
**Age (y)**	18.9 ± 0.8 19.0 (18.0; 19.8)	25.0 ± 3.5 24.0 (21.0; 28.0)	22.4 ± 4.1 21.0 (19.0; 26.0)
**Height (cm)**	168.9 ± 3.2 169.5 (166.0; 171.0)	172.4 ± 7.3 171.0 (168.0; 177.0)	170.9 ± 6.1 170.5 (166.0; 172.0)
**Weight (kg)**	61.2 ± 4,7 60.8 (56.4; 64.3)	67.3 ± 6.5 66.1 (64.1; 73.3)	64.9 ± 6.5 64.7 (59.5; 69.6)
**BMI (kg/m^2^)**	21.9 ± 2.1 20.9 (19.6; 23.8)	22.8 ± 1.6 22.3 (21.8; 23.8)	22.4 ± 1.8 22.3 (20.6; 23.8)
**Training hours**	10.9 ± 1.2 11.0 (10.0; 11.0)	16.7 ± 2.4 18.0 (14.0; 18.0)	14.3 ± 3.6 14.0 (11.0; 18.0)
**1RM_est_ for the back squat (kg)**	101.7 ± 18.6 96.5 (75.6; 141.7)	118.0 ± 19.8 110.2 (93.0; 153.3)	110.8 ± 20.9 106.8 (75.6; 153.3)
**ROM for the back squat (cm)**	45.3 ± 7.3 42.5 (38.0; 59.0)	62.2 ± 6.1 61.0 (54.0; 77.0)	54.7 ± 10.8 57.5 (38.0; 77.0)

Abbreviations: 1RM_est_ = estimated one-repetition maximum; ROM = range of motion; SD = standard deviation.

**Table 4:** Hormonal changes 60 min post-training (adjusted by means of hematocrit).

Parameter [nmol/L] [µmol/L] (Marked with #)	Pre	Post	Absolute Changes	P*wil* [pt] Effect Size	Relative Changes	P*wil* [pt] Effect Size
	Mean (SD) Median	Mean (SD) Median	Mean (SD) Median		Mean (SD) Median	
**11-Deoxycorticosterone** (11-DOC)	0.084 (0.040) 0.089	0.061 (0.031) 0.089	−0.023 (0.027) −0.011	0.001 [0.001] 0.86	77.7% (29.1%) 80.6%	0.002 [0.004] 0.77
**11-Deoxycortisol** (S)	0.530 (0.241) 0.568	0.254 (0.184) 0.205)	−0.275 (0.270) −0.206	<0.001 [<0.001] 1.02	53.5% (34.7%) 45.3%	<0.001 [<0.001] 1.34
**11-Ketoandrostenedione** (11KA4)	0.414 (0.160) 0.369	0.493 (0.185) 0.476	0.079 (0.139) 0.081	0.023 [0.023] 0.57	124.3% (45.0%) 120.3%	0.023 [0.030] 0.54
**11β-Hydroxyandrostenedione (11OHA4)**	3.122 (1.052) 2.990	2.415 (1.139) 1.883	−0.707 (1.083) −0.589	0.012 [0.011] 0.65	79.9% (31.5%) 76.7%	0.011 [0.013] 0.64
**16α-Hydroxyprogesterone** (16αOHP4)	0.257 (0.121) 0.240	0.175 (0.082) 0.156	−0.082 (0.120) −0.053	0.002 [0.008] 0.69	78.0% (34.4%) 76.2%	0.009 [0.012] 0.64
**21-Deoxycortisol** (21DF)	0.043 (0.042) 0.028	0.010 (0.011) 0.008	−0.032 (0.040) −0.021	<0.001 [0.003] 0.80	37.2% (46.2%) 19.2%	<0.001 [<0.001] 1.36
**Androsterone** (AST)	0.758 (0.594) 0.697	0.557 (0.274) 0.478	−0.201 (0.582) −0.045	0.049 [0.150] 0.34	85.2% (29.1%) 93.1%	0.045 [0.039] 0.51
**Corticosterone** (CORT)	9.059 (4.883) 8.739	4.052 (3.260) 3.022	−5.007 (5.763) −6.146	0.001 [0.001] 0.87	56.9% (46.5%) 37.2%	0.001 [0.001] 0.93
**Cortisol** # (F)	0.309 (0.093) 0.288	0.206 (0.075) 0.208	−0.102 (0.083) −0.122	<0.001 [<0.001] 1.22	69.1% (24.0%) 67.1%	<0.001 [<0.001] 1.28
**Dehydroepiandrosterone** (DHEA)	19.460 (7.793) 18.515	15.647 (5.485) 16.094	−3.813 (6.342) −1.909	0.006 [0.017] 0.60	82.9% (22.2%) 89.5%	0.006 [0.004] 0.77
**Dehydroepiandrosteronsulfate** # (DHEAS)	6847.7 (4279.5) 5700.9	7198.4 (3886.5) 6216.2	350.7 (1580.8) 488.5	0.145 [0.346] 0.22	109.9% (19.0%) 107.0%	0.045 [0.036] 0.52
**Testosterone** (T)	0.771 (0.383) 0.674	0.726 (0.401) −0.042	−0.046 (0.111) −0.042	0.080 [0.089] 0.41	93.8% 16.5%) 92.9%	0.080 [0.119] 0.38
**Profile (arithmetic sum)** **[nmol/L]**	**Mean** **(SD)** **Median**	**P*wil*** **[pt]**	**Effect** **size**	**Mean** **(SD)** **Median**	**P*wil*** **[pt]**	**Effect size**
**Androgenic hormones** (11OHA4, 11OHT, 11KA4, 11KT, DHT, A4, AST, DHEA, DHEAS, T, ETIO)	345.7 (1582.5) 483.9	0.145 [0.354]	0.22	109.7% (18.9%) 107.0%	0.055 [0.038]	0.51
**Adrenal androgenic hormones** (11KA4, 11KT, 11OHA4, 11OHT, DHEAS)	349.8 (1580.8) 486.0	0.145 [0.348]	0.22	109.8% (19.0%) 107.0%	0.045 [0.037]	0.52
**Glucocorticoids** (S, 21DF, F, E)	−107.39 (88.09) −134.46	<0.001 [<0.001]	1.22	71.8% (22.8%) 74.8%	<0.001 [<0.001]	1.23
**Mineralocorticoids** (11-DOC, CORT, ALDO)	−5.058 (5.841) −6.172	0.001 [0.001]	0.87	58.5% (45.5%) 39.4%	0.001 [0.001]	0.91
**Classic androgen pathway** (DHT, A4, AST, DHEA, ETIO)	−4.515 (6.861) −2.622	0.005 [0.010]	0.66	84.6% (19.7%) 84.1%	0.005 [0.003]	0.78
**11-oxy pathway** (11OHA4, 11OHT, 11KA4, 11KT, A4, T)	−0.787 (2.242) −1.266	0.096 [0.143]	0.35	92.3% (27.3%) 85.7%	0.113 [0.143]	0.28
**Backdoor pathway** (17OHP4, DHT, AST, P4)	0.040 (2.000) 0.072	0.953 [0.932]	0.02	98.9% (23.0%) 101.5%	0.922 [0.835]	0.05
**Weighted profile of the classic androgen pathway**	−1.000 (0.582) 0.985	<0.001 [<0.001]	1.72			
**Weighted profile of the 11-oxy pathway**	−1.000 (1.032) −1.232	0.001 [0.001]	0.97			
**Weighted profile of the backdoor pathway**	−0.999 (1.390) −0.818	0.007 [0.006]	0.72			

Abbreviations: 11-DOC = 11-Deoxycorticosterone; S = 11-Deoxycortisol; 11KA4 = 11-Ketoandrostenedione; 11KT = 11-Ketotestosterone; 11OHA4 = 11β-Hydroxyandrostenedione; 11OHT = 11β-Hydroxytestosterone; 16αOHP4 = 16α-Hydroxyprogesterone; 17α20α-diOHP4 = 17α,20α-Dihydroxyprogesterone; 17OHP4 = 17α-Hydroxyprogesterone; 21DF = 21-Deoxycortisol; DHT = 5α-Dihydrotestosterone; ALDO = Aldosterone; A4 = Androstenedione; AST = Androsterone; E = Cortisone; CORT = Corticosterone; F = Cortisol; DHEA = Dehydroepiandrosterone; DHEAS = Dehydroepiandrosteronsulfate; ETIO = Etiocholanolone; P5 = Pregnenolone; P4 = Progesterone; T = Testosterone.

## Data Availability

The original contributions presented in the study are included in the article/Supplementary Materials, further inquiries can be directed to the corresponding author/s.

## Supplementary Materials

The following supporting information can be downloaded at https://www.mdpi.com/article/10.3390/sports13120426/s1, Table S1: Hormonal changes 60 min post-training (adjusted by means of hematocrit); Table S2: Change in pregnenolone concentrations across visits (1-4) in relation to the menstrual cycle phase (defined by progesterone concentration); Supplementary Material File S1: Explanation of the weighted profile.
