# Supplementary material for: Acute Adrenal Suppression Following Resistance Training in Elite Female Athletes: A Comprehensive Steroid Profile
Source: Sports (Basel). 2025 Dec 3;13(12):426. doi: 10.3390/sports13120426 (PMC12737264; doi:10.3390/sports13120426)
Supplement: Supplementary file 1 [file sports-13-00426-s001.zip › Supplemental Table S1_Hormonal changes 60 minutes post-training_long list.pdf]

Supplemental Table S1. Hormonal changes 60 min post-training (adjusted by means of hematocrit):

Parameter<br>[nmol/l]<br>[µmol/l] (marked with #)	Pre	Post	Absolute changes	P*wil*<br>[pt]<br>Effect size	Relative changes	P*wil*<br>[pt]<br>Effect size
	Mean<br>(SD)<br>Median	Mean<br>(SD)<br>Median	Mean<br>(SD)<br>Median		Mean<br>(SD)<br>Median	
**11-Deoxycorticosterone**<br>(11-DOC)	0.084<br>(0.040)<br>0.089	0.061<br>(0.031)<br>0.089	-0.023<br>(0.027)<br>-0.011	0.001<br>[0.001]<br>0.856	77.7%<br>(29.1%)<br>80.6%	0.002<br>[0.004]<br>0.768
**11-Deoxycortisol**<br>(S)	0.530<br>(0.241)<br>0.568	0.254<br>(0.184)<br>0.205)	-0.275<br>(0.270)<br>-0.206	< 0.001<br>[< 0.001]<br>1.021	53.5%<br>(34.7%)<br>45.3%	< 0.001<br>[< 0.001]<br>1.339
**11-Ketoandrostenedione**<br>(11KA4)	0.414<br>(0.160)<br>0.369	0.493<br>(0.185)<br>0.476	0.079<br>(0.139)<br>0.081	0.023<br>[0.023]<br>0.571	124.3%<br>(45.0%)<br>120.3%	0.023<br>[0.030]<br>0.542
**11-Ketotestosterone**<br>(11KT)	1.220<br>(0.465)<br>1.184	1.334<br>(0.711)<br>1.221	0.114<br>(0.492)<br>0.023	0.651<br>[0.326]<br>0.232	108.8%<br>(39.3%)<br>101.9%	0.515<br>[0.341]<br>0.224
**11β-Hydroxyandrostenedione**<br>(11OHA4)	3.122<br>(1.052)<br>2.990	2.415<br>(1.139)<br>1.883	-0.707<br>(1.083)<br>-0.589	0.012<br>[0.011]<br>0.653	79.9%<br>(31.5%)<br>76.7%	0.011<br>[0.013]<br>0.636
**11β-Hydroxytestosterone**<br>(11OHT)	0.228<br>(0.096)<br>0.214	0.260<br>(0.162)<br>0.188	0.032<br>(0.121)<br>0.015	0.352<br>[0.258]<br>0.268	114.5%<br>(52.5%)<br>110.8%	0.465<br>[0.243]<br>0.277
**16α-Hydroxyprogesterone**<br>(16αOHP4)	0.257<br>(0.121)<br>0.240	0.175<br>(0.082)<br>0.156	-0.082<br>(0.120)<br>-0.053	0.002<br>[0.008]<br>0.685	78.0%<br>(34.4%)<br>76.2%	0.009<br>[0.012]<br>0.640
**17α,20α-Dihydroxyprogesterone**<br>(17α20α-diOHP4)	0.625<br>(0.220)<br>0.643	0.600<br>(0.243)<br>0.653	-0.025<br>(0.153)<br>-0.022	0.312<br>[0.486]<br>0.163	96.6%<br>(24.7%)<br>91.2%	0.595<br>[0.552]<br>0.139
**20α-Hydroxyprogesterone**<br>(20α-OHP)	1.008<br>(0.875)<br>0.718	1.111<br>(1.019)<br>0.743	0.103<br>(0.245)<br>0.039	0.096<br>[0.083]<br>0.421	106.1%<br>(14.1%)<br>108.3%	0.055<br>[0.077]<br>0.430
**17α-Hydroxyprogesterone**<br>(17OHP4)	1.461<br>(0.661)<br>1.461	1.321<br>(0.640)<br>1.414	-0.140<br>(0.381)<br>-0.151	0.241<br>[0.126]<br>0.338	91.6%<br>(30.2%)<br>91.8%	0.312<br>[0.242]<br>0.278
**21-Deoxycortisol**<br>(21DF)	0.043<br>(0.042)<br>0.028	0.010<br>(0.011)<br>0.008	-0.032<br>(0.040)<br>-0.021	< 0.001<br>[0.003]<br>0.802	37.2%<br>(46.2%)<br>19.2%	< 0.001<br>[< 0.001]<br>1.359
**5α-Dihydrotestosterone**<br>(5DHT)	0.554<br>(0.256)<br>0.463	0.488<br>(0.237)<br>0.469	-0.067<br>(0.208)<br>-0.050	0.275<br>[0.181]<br>0.319	90.0%<br>(25.3%)<br>91.4%	0.169<br>[0.102]<br>0.396

**Aldosterone**<br>(ALDO)	0.275<br>(0.183)<br>0.233	0.247<br>(0.118)<br>0.277	-0.028<br>(0.154)<br>0.002	0.776<br>[0.435]<br>0.183	108.7%<br>(54.0%)<br>100.3%	0.623<br>[0.493]<br>0.164
**Androstenedione**<br>(A4)	3.098<br>(1.074)<br>2.846	2.839<br>(1.208)<br>2.807	-0.260<br>(0.557)<br>-0.245	0.080<br>[0.057]<br>0.466	91.9%<br>(19.0%)<br>89.3%	0.080<br>[0.079]<br>0.426
**Androsterone**<br>(AST)	0.758<br>(0.594)<br>0.697	0.557<br>(0.274)<br>0.478	-0.201<br>(0.582)<br>-0.045	0.049<br>[0.150]<br>0.345	85.2%<br>(29.1%)<br>93.1%	0.045<br>[0.039]<br>0.510
**Cortisone**<br>(E)	46.993<br>(8.902)<br>46.620	42.629<br>(12.292)<br>39.617	-4.364<br>(10.318)<br>-1.731	0.123<br>[0.082]<br>0.423	91.3%<br>(21.6%)<br>96.5%	0.113<br>[0.096]<br>0.404
**Corticosterone**<br>(CORT)	9.059<br>(4.883)<br>8.739	4.052<br>(3.260)<br>3.022	-5.007<br>(5.763)<br>-6.146	0.001<br>[0.001]<br>0.869	56.9%<br>(46.5%)<br>37.2%	0.001<br>[0.001]<br>0.926
**Cortisol**<br>(F)#	0.309<br>(0.093)<br>0.288	0.206<br>(0.075)<br>0.208	-0.102<br>(0.083)<br>-0.122	< 0.001<br>[< 0.001]<br>1.224	69.1%<br>(24.0%)<br>67.1%	< 0.001<br>[< 0.001]<br>1.287
**Dehydroepiandrosterone**<br>(DHEA)	19.460<br>(7.793)<br>18.515	15.647<br>(5.485)<br>16.094	-3.813<br>(6.342)<br>-1.909	0.006<br>[0.017]<br>0.601	82.9%<br>(22.2%)<br>89.5%	0.006<br>[0.004]<br>0.770
**Dehydroepiandrosteronsulfate**<br>(DHEAS)#	6.847<br>(4.279)<br>5.700	7.198<br>(3.886)<br>6.216	0.350<br>(1.580)<br>0.488	0.145<br>[0.346]<br>0.222	109.9%<br>(19.0%)<br>107.0%	0.045<br>[0.036]<br>0.518
**Etiocholanolone**<br>(ETIO)	0.961<br>(0.718)<br>0.670	0.832<br>(0.596)<br>0.700	-0.129<br>(0.444)<br>-0.073	0.490<br>[0.221]<br>0.291	96.1%<br>(32.8%)<br>85.8%	0.395<br>[0.609]<br>0.120
**Pregnenolone**<br>(P5)	1.010<br>(0.901)<br>1.060	0.980<br>(0.893)<br>0.771	-0.116<br>(0.736)<br>-0.092	0.651<br>[0.502]<br>0.157	129.7%<br>(112.3%)<br>94.1%	0.615<br>[0.264]<br>0.265
**Progesterone**<br>(P4)	4.119<br>(4.022)<br>2.396	4.625<br>(4.731)<br>2.685	0.506<br>(1.420)<br>0.013	0.293<br>[0.138]<br>0.356	105.7%<br>(23.6%)<br>106.0%	0.293<br>[0.304]<br>0.242
**Testosterone**<br>(T)	0.771<br>(0.383)<br>0.674	0.726<br>(0.401)<br>-0.042	-0.046<br>(0.111)<br>-0.042	0.080<br>[0.089]<br>0.412	93.8%<br>16.5%)<br>92.9%	0.080<br>[0.119]<br>0.376

**Profile (arithmetic sum) [nmol/l]**	**Mean (SD) Median**	***Pwil* [pt]**	**Effect size**	**Mean (SD) Median**	***Pwil* [pt]**	**Effect size**
**Androgenic hormones** (11OHA4, 11OHT, 11KA4, 11KT, DHT, A4, AST, DHEA, DHEAS, T and ETIO)#	0.345<br>(1.582)<br>0.483	0.145<br>[0.354]	0.218	109.7%<br>(18.9%)<br>107.0%	0.055<br>[0.038]	0.513

**Adrenal androgenic hormones** (11KA4, 11KT, 11OHA4, 11OHT, DHEAS)#	0.349<br>(1.580)<br>0.486	0.145<br>[0.348]	0.221	109.8%<br>(19.0%)<br>107.0%	0.045<br>[0.037]	0.518
**Glucocorticoids** (S, 21DF, F, E)#	-0.107<br>(0.088)<br>-0.134	< 0.001<br>[< 0.001]	1.222	71.8%<br>(22.8%)<br>74.8%	< 0.001<br>[< 0.001]	1.233
**Mineralocorticoids** (11-DOC, CORT, ALDO)	-5.058<br>(5.841)<br>-6.172	0.001<br>[0.001]	0.866	58.5%<br>(45.5%)<br>39.4%	0.001<br>[0.001]	0.912
**Classic androgen pathway** (DHT, A4, AST, DHEA, ETIO)	-4.515<br>(6.861)<br>-2.622	0.005<br>[0.010]	0.6658	84.6%<br>(19.7%)<br>84.1%	0.005<br>[0.003]	0.783
**11-oxy pathway** (11OHA4, 11OHT, 11KA4, 11KT, A4 and T)	-0.787<br>(2.242)<br>-1.266	0.096<br>[0.143]	0.351	92.3%<br>(27.3%)<br>85.7%	0.113<br>[0.237]	0.281
**Backdoor pathway** (17OHP4, DHT, AST, P4)	0.040<br>(2.000)<br>0.072	0.953<br>[0.932]	0.020	98.9%<br>(23.0%)<br>101.5%	0.922<br>[0.835]	0.048
**Weighted profile of the classic androgen pathway**	-1.000<br>(0.582)<br>0.985	<0.001<br>[<0.001]	1.690			
**Weighted profile of the 11-oxy pathway**	-1.000<br>(1.032)<br>-1.232	0.001<br>[0.001]	0.946			
**Weighted profile of the backdoor pathway**	-0.999<br>(1.390)<br>-0.818	0.007<br>[0.006]	0.719			

Abbreviations: 11-DOC = 11-Deoxycorticosterone; S= 11-Deoxycortisol, 11KA4 = 11-Ketoandrostenedione; 11KT = 11-Ketotestosterone; 11OHA4 = 11β-Hydroxyandrostenedione; 11OHT = 11β-Hydroxytestosterone; 16αOHP4 = 16α-Hydroxyprogesterone; 17α20α-diOHP4 = 17α,20α-Dihydroxyprogesterone; 17OHP4 = 17α-Hydroxyprogesterone; 21DF = 21-Deoxycortisol; DHT = 5α-Dihydrotestosterone; ALDO = Aldosterone; A4 = Androstenedione; AST = Androsterone; E = Cortisone; CORT = Corticosterone; F = Cortisol; DHEA = Dehydroepiandrosterone; DHEAS = Dehydroepiandrosteronsulfate; ETIO = Etiocholanolone; P5 = Pregnenolone; P4 = Progesterone; T = Testosterone.

.
