# Supplementary material for: Acute Adrenal Suppression Following Resistance Training in Elite Female Athletes: A Comprehensive Steroid Profile
Source: Sports (Basel). 2025 Dec 3;13(12):426. doi: 10.3390/sports13120426 (PMC12737264; doi:10.3390/sports13120426)
Supplement: Supplementary file 1 [file sports-13-00426-s001.zip › Supplemental Table S2_Change in P5 across the MC.pdf]

Supplemental Table S2: Change in pregnenolone concentrations across visits (1-4) in relation to the menstrual cycle phase (defined by progesterone concentration)

Subgroup P1 (follicular phase): Concentration of progesterone at visit (pre) < 1 nmol/l (N = 15)
Subgroup P2 (luteal phase): Concentration of progesterone at visit (pre) > 5 nmol/l (N = 2)

Change in Pregnenolone [nmol/l]	Difference post-pre (absolute)					Changes post/pre (relative)		
	P1 N	P2 N	P1 Mean (SD)	P2 Mean (SD)	p_{wil} [p_t]	P1 Mean (SD)	P2 Mean (SD)	p_{wil} [p_t]
**Visit 1**	15	2	-0.299 (0.947)	1.305 (0.727)	0.038 [0.041]	245.5% (599.1%)	156.5% (40.3%)	0.258 [0.844]
**Visit 2**	10	4	-0.061 (1.562)	1.236 (2.157)	0.304 [0.229]	461.6% (1317.1%)	468.9% (442.6%)	0.046 (1) [0.992]
**Visit 3**	8	2	-0.012 (1.015)	0.640(2)	0.489 [0.434]	151.9% (258.0%)	298.3% (2)	0.667 [0.632]
**Visit 4**	8	3	-0.740 (2.036)	0.683 (1.671)	0.921 [0.312]	303.4% (589.0%)	117.7% (139.8%)	1.000 [0.621]

(1) A lot of post-values were, that's why the analysis of relative changes can be distorted.
(2) Due to a pre-value was zero the relative change is calculated only for N = 1.
