# Supplementary material for: Acute Adrenal Suppression Following Resistance Training in Elite Female Athletes: A Comprehensive Steroid Profile
Source: Sports (Basel). 2025 Dec 3;13(12):426. doi: 10.3390/sports13120426 (PMC12737264; doi:10.3390/sports13120426)
Supplement: Supplementary file 1 [file sports-13-00426-s001.zip › Supplementary Material File S1_Explanation of the weighted profile.pdf]

Supplementary Material S1: Detailed explanation and regression model of the "weighted profile"

This hormone profile is defined by the sum of the concentrations of the 6 hormones (see table below). The summation does not result in any increase in performance, particularly due to the different directions of the changes.

	Absolute changes [nmol/l]			Relative changes [%]		
**Parameter/Profile**	**Mean (SD) Median**	**Pwil [pt]**	**Effect size**	**Mean (SD) Median**	**Pwil [pt]**	**Effect size**
**11b-Hydroxy-Androstenedione** (11OHA4)	-0.707 (1.083) -0.589	0.012 [0.011]	0.65	79.9% (31.5%) 76.7%	0.011 [0.013]	0.64
**11-Hydroxy-testosterone** (11OHT)	0.032 (0.121) 0.015	0.352 [0.258]	0.27	114.5% (52.5%) 110.8%	0.465 [0.243]	0.28
**11-Ketoandro-stenedione** (11KA4)	0.079 (0.139) 0.081	0.023 [0.023]	0.57	124.3% (45.0%) 120.3%	0.023 [0.030]	0.54
**11-Keto-Testosterone** (11KT)	0.114 (0.492) 0.023	0.651 [0.326]	0.23	108.8% (39.3%) 101.9%	0.515 [0.341]	0.22
**Androstenedione** (A4)	-0.260 (0.557) -0.245	0.080 [0.057]	0.53	85.2% (29.1%) 93.1%	0.045 [0.039]	0.43
**Testosterone** (T)	-0.046 (0.111) -0.042	0.080 [0.089]	0.41	93.8% (16.5%) 92.9%	0.080 [0.119]	0.38
**Profile 11-oxy** pathway	-0.787 (2.242) -1.266	0.096 [0.143]	0.35	92.3% (27.3%) 85.7%	0.113 [0.143]	0.28

Table 1: Overview about changes in hormone profile 11-oxy pathway

The use of the arithmetic means of the individual relative changes (102.2% ± 31.3%; Median 93.8%) or their geometric mean (99.8% ± 30.4%; Median 92.1%) confirms the result of relative change. Because of the different sizes, though, the (simple) sum is mostly influenced by individual hormones. In particular, the concentration of 11b-Hydroxyandrostenedione has a statistically significant correlation ($r = 0.946$; $p_{SR} < 0.001$) with this simple sum.

In addition, the concentrations of the hormones correlate with each other.

Correlation Spearman-Rho: Coefficient/p value					
	**11OHT**	**11KA4**	**11KT**	**A4**	**T**
**11OHA4**	0.530/0.020	0.581/0.009	0.658/0.002	0.705/0.001	0.621/0.005
**11OHT**	-	0.602/0.006	0.826/<0.001	0.544/0.016	0.735/<0.001
**11KA4**	-	-	0.784/<0.001	0.539/0.017	0.612/0.005
**11KT**	-	-	-	0.618/0.005	0.786/<0.001
**A4**	-	-	-	-	0.872/<0.001

Table 2: Non-parametric correlation within 11-oxy-pathway

To compensate for differences in size, linear regression models are applied to the hormone concentrations based on the (normalized1) concentration of 11OHA4.

**Change in 11b-Hydroxyandrostenedione = B x (Changes in …) + C**	**Linear Regression Model**				
	**Coefficient B Hormone**	**p**	**Coefficient C Constant**	**p**	**R^2**
**11-Hydroxytestosterone**	6.054	0.001	-0.903	< 0.001	0.677
**11-Ketoandro-stenedione**	5.493	0.001	-1.142	< 0.001	0.704
**11-Ketotestosterone**	1.630	< 0.001	-0.893	< 0.001	0.741
**Androstenedione**	1.436	< 0.001	-0.334	0.099	0.738
**Testosterone**	6.856	0.001	-0.395	0.063	0.700

Table 3: Coefficients of the linear regression model (11-oxy pathway)

Conversion using linear regression models results in the mean change for all concentrations of hormones and also for the mean of these weighted changes being the same -1.000.

	Absolute changes [nmol/l]		
	Mean (SD) Median	Pwil [pt]	Effect size
Weighted 11-oxy pathway	-1.000 (1.032) -1.232	0.001 [0.001]	0.97

Table 4: Overview about changes in hormone profile (11-oxy pathway, weighted)

1 divided by the absolute value of the mean (0.707)
